# Human T Cell and Antibody-Mediated Responses to the *Mycobacterium tuberculosis* Recombinant 85A, 85B, and ESAT-6 Antigens

**DOI:** 10.1155/2011/351573

**Published:** 2010-12-29

**Authors:** Gilson C. Macedo, Adriana Bozzi, Helena Rachel Weinreich, Andre Bafica, Henrique C. Teixeira, Sergio C. Oliveira

**Affiliations:** ^1^Laboratory of Immunology of Infectious Diseases, Department of Biochemistry and Immunology, Institute of Biological Sciences, Federal University of Minas Gerais, 31270-901 Belo Horizonte, MG, Brazil; ^2^Department of Parasitology, Microbiology and Immunology, Biological Sciences Institute, Federal University of Juiz de Fora, 36036-900 Juiz de Fora, MG, Brazil; ^3^Oswaldo Cruz Health Center, Belo Horizonte-Minas Gerais, 30180-080 Belo Horizonte, MG, Brazil; ^4^Department of Microbiology, Immunology and Parasitology, Federal University of Santa Catarina, 88040-900 Florianopolis, MG, Brazil

## Abstract

Tuberculosis remains a major health problem throughout the world causing large number of deaths. Effective disease control and eradication programs require the identification of major antigens recognized by the protective responses against *M. tuberculosis*. In this study, we have investigated humoral and cellular immune responses to *M. tuberculosis*-specific Ag85A, Ag85B, and ESAT-6 antigens in Brazilian patients with pulmonary (P, *n* = 13) or extrapulmonary (EP, *n* = 12) tuberculosis, patients undergoing chemotherapy (PT, *n* = 23), and noninfected healthy individuals (NI, *n* = 7). Compared to NI, we observed increased levels of IgG1 responses to Ag85B and ESAT-6 in P and PT groups. Regarding cellular immunity, Ag85A and ESAT-6 were able to discriminate P, PT, and EP patients from healthy individuals by IFN-*γ* production and P and PT groups from EP individuals by production of TNF-*α*. In summary, these findings demonstrate the ability of Ag85A, Ag85B, and ESAT-6 to differentiate TB patients from controls by IgG1, IFN-*γ* and TNF-*α* production.

## 1. Introduction

Tuberculosis (TB) remains the largest single infectious cause of death globally. It is estimated that 30% of the word population is infected with *Mycobacterium tuberculosis* resulting in approximately 2-3 million deaths each year [1]. Further, the AIDS epidemic and the appearance of multidrug resistant strains of *M. tuberculosis* have contributed to the reemergence of TB in developing countries; however, this disease continues to be a devastating entity in the developing world [2]. At present, the only registered vaccine against tuberculosis, *Mycobacterium bovis *Bacillus Calmette-Guérin (BCG), was introduced in 1921 and has been widely used; however, its effectiveness remains controversial because their protection levels are extremely variable in different population [3–5]. Furthermore, vaccination with *M. bovis* BCG is contraindicated in immunocompromised subjects, including acquired immunodeficiency syndrome patients, who are usually at a very high risk of developing TB [6]. In addition, the diagnostic value of the presently used skin test reagent, purified protein derivative (PPD) of *Mycobacterium tuberculosis*, is low, owing to cross-reactivity with environmental mycobacteria and vaccine strains of *M. bovis* BCG [7, 8]. Thus, the effective control and eradication of TB is dependent upon the availability of effective vaccines and reagents for specific diagnosis. For this purpose, the identification of major antigens recognized by the protective immune response against *M. tuberculosis* remains a critical step.

Among *M. tuberculosis* antigens studied, the 30/32 KDa antigen 85 (Ag85) complex has been the focus of intense research over the past several years and comprises three closely related proteins, 85A (32 KDa), 85B (30 KDa), and 85C (32.5 KDa) that possess enzymatic mycolyl-transferase activity [9–11]. The Ag85 complex induces protective immunity against TB in guinea pigs [12], and strong proliferation and IFN-*γ* production in peripheral blood mononuclear cells (PBMC) from healthy tuberculin reactors [13]. Regarding, ESAT-6, the early secreted antigenic target is a low-molecular-weight protein essentially present in pathogenic mycobacteria including members of the mycobacterium complex (*M. tuberculosis, M bovis,* and *M. africanum*) and *M. leprae* [14]. Analysis of T-cell responses to *M. tuberculosis* ESAT-6 showed an elevated range of recognition from many tuberculosis patients [15]. Consequently, the possible use of ESAT-6 as a marker of *M. tuberculosis* infection has been proposed. Moreover, other studies have demonstrated the ability of this protein to discriminate tuberculosis patients from health donors in a high endemic area [16]. Additionally, ESAT-6 is able to differentiate tuberculosis patients from both BCG-vaccinated individuals and *M. avium* infected patients [17]. 

The main goal of this study was to evaluate the cellular and humoral immune responses to the recombinant proteins Ag85A, Ag85B, and ESAT-6 in Brazilian pulmonary and extra-pulomary tuberculosis patients and individuals undergoing chemotherapy. The recombinant proteins were produced in *E. coli* and purified by affinity chromatography. Cellular proliferation and cytokine production were evaluated in peripheral blood mononuclear cells (PBMC) and specific antibody isotypes to Ag85A, Ag85B and ESAT-6 were measured in serum of TB patients and controls. In this study, we have shown the ability of Ag85B and ESAT-6 to differentiate TB patients from controls by IgG1 production. Additionally, the results here demonstrated that Ag85A and ESAT-6 were able to discriminate P, PT, and EP patients from healthy individuals by IFN-*γ* production and P and PT groups from EP individuals by production of TNF-*α*.

## 2. Materials and Methods

### 2.1. Study Population

Patients with active pulmonary TB (P, *n* = 13) or active extra-pulmonary TB (EP, *n* = 12), and pulmonary TB patients with 1–3 months of anti-TB chemotherapy (PT, *n* = 23), diagnosed at the outpatient unit of the Oswaldo Cruz Health Center, Belo Horizonte, Minas Gerais, Brazil, were enrolled in this study. All TB patients had sputum-positive bacilloscopy or culture-confirmed disease. The EP-TB group comprised six pleural TB, five miliary TB and one intestinal TB as shown in Table 1. Seven healthy non-BCG vaccinated individuals (all PPD-) without prior history of mycobacterial infection were included as control group. All enrolled patients tested negative by ELISA for HIV. None of the individuals had evidence of acute infections (other than TB) at the time of sample collection. Twenty ml of blood was taken from each patient.

### 2.2. Ethics Committee

All patients gave permission for blood sampling after written consent, and the Ethics Committee of the Santa Casa Hospital at Belo Horizonte, Minas Gerais, Brazil approved the research protocol.

### 2.3. Mycobacterial Antigens

The recombinant Ag85A, Ag85B e ESAT-6 was produced using the pMAL-c2 expression system. Briefly, the pMAL-85A, pMAL-85B, or pMAL-ESAT-6 construct was used to transform *Escherichia coli* DH5*α* strain as previously described [18]. Bacterial cells were induced using 0.42 mM IPTG (isopropyl-*β*-D-thiogalactoside) and recombinant proteins fused to the maltose binding protein (MBP) were produced. Three hours after gene expression, the cells were harvested and lysed using thermal shock, sonication, and lysozyme treatment. The fusion protein recovered in the supernatant was then purified by affinity chromatography using an amylose resin (New England BioLabs). Residual endotoxin levels were removed from recombinant proteins by using Triton X-114 and measured to be <50 EU/mg recombinant protein by the LAL assay (Limulus amoebocyte lysate). Purified protein derivative (PPD-RT50) was obtained from Statens Serum Institute, Copenhagen, Denmark.

### 2.4. T Cell Proliferation Assays

Heparinized venous blood was obtained from all patients and controls, and peripheral blood mononuclear cells (PBMCs) were isolated by Ficoll-Hypaque (Ficoll 6,42% (SIGMA) and Hypaque 50% (Sanofi Synthelab)) density centrifugation. Cells were washed in RPMI 1640 fresh medium and cultured (2,5 × 10^5^ cells/well) in flat-bottom 96-well plates (Nunc Brand Products) in 200 *μ*L of RPMI 1640 medium supplemented with 10% AB + heat-inactivated human serum and 1% antibiotic/antimycotic (Gibco-BRL) and incubated at 37°C in a humidified 5% CO_2_ incubator with recombinant antigens or medium alone (control). The recombinant antigens were titered to determine the optimal protein concentration for proliferation assays. Ag85A, Ag85B, and PPD was used at 25 *μ*g/ml, ESAT-6 at 50 *μ*g/ml, and phytohaemagglutinin (PHA) was used at 10 *μ*g/ml. The concentration of the recombinant antigens tested here was higher compared to the used by other authors because we produced the mycobacterial antigens fused to MBP that has itself a molecular mass of approximately 42.6 kDa. All antigens were plated in triplicate. After 3 days (mitogen) or 5 days (antigens) of incubation, [^3^H] thymidine (0.5 *μ*Ci/well) was added to the cultures. Eighteen hours later, the supernatants were collected, and the cells were harvested. The incorporated radioactivity in the cells was evaluated by liquid scintillation spectroscopy as a measure of cellular proliferation. Mean counts per minute for triplicate cultures and stimulation index (SI) were obtained for each patient. The SI was the ratio of mean counts per minute in the presence of antigen to means counts per minute in medium alone.

### 2.5. Cytokine Measurement

Concentration of human IFN-*γ*, TNF-*α*, IL-10, and IL-4 in cell culture supernatants of proliferation assays was determined by enzyme-linked immunosorbent assay (ELISA) using kits Duoset from R&D Systems (Minneapolis, MN, USA) according to manufacturer's directions.

### 2.6. Detection of Antibody Responses

Detection of antibody against recombinant Ag85A, Ag85B, and ESAT-6 antigens in TB patients and healthy individual sera was performed by a modified ELISA [19]. Briefly, ninety-six-well flat-bottom microtiter plates (Nunc, Roskilde, Denmark) were coated overnight at 4°C with 100 *μ*L of each recombinant antigen separately at a concentration of 5 *μ*g/ml in 0.1 M carbonate bicarbonate buffer (pH 9.6) per well. The plates were then blocked with 10% bovine fetal serum in PBS (pH 7.4) for 2 h at room temperature. Subsequently, the plates were washed three times with PBS plus 0.05% Tween-20 (PBS-T20). Serum samples diluted 1 : 100 in PBS-T20 (100 *μ*L/well) were added in duplicate, and the plates were incubated for 1 h at room temperature. Peroxidase-labeled anti-IgG (Sigma Chemical Co., St. Louis, MO), anti-IgM (Sigma), and anti-IgA (Sigma) were added at dilutions of 1 : 2,000, 1 : 2,000, and 1 : 10,000 (100 *μ*L/well), respectively. After 1 h at 37°C, the plates were washed, and OPD (orthophenyl-diaminobenzidine) plus 0.05% hydrogen peroxide in phosphate citrate buffer (pH 5) was added (100 *μ*L/well). This mixture was then incubated for 30 min at room temperature, and the reaction was stopped by addition of 5% H_2 _SO_4_ (50 *μ*L/well). Absorbance was read at 492 nm using a microplate reader (BioRad, Hercules, CA). To determine IgG subclasses levels, the previous protocol was slightly modified. The serum dilution was changed to 1:80 for IgG1 and IgG3 and 1:20 to IgG2 and IgG4. Diluted sera were added to the plates, and they were incubated for 2 h at 37°C. After washing, peroxidase-labeled antihuman antibody was dispensed in each well at concentrations of 1 : 1,000 (IgG1, IgG3) or 1 : 500 (IgG2, IgG4), and the plates were incubated for 12–16 h at 4°C. The next steps were identical to those described above.

### 2.7. Statistical Analysis

Results are reported as means ± standard errors. Differences between responses from TB patient groups and control groups were analyzed with nonparametric Kruskall-Wallis test. Correlation between ESAT-6 induced IFN-*γ* and proliferation responses from patients WAS identified using Spearman Correlation. Statistical analysis was performed using the GraphPad Prism software version 5.0 (GraphPad software incorporated). Statistical differences were considered significant at *P* < .05.

## 3. Results

### 3.1. IgG1 Is the Predominant Antibody Isotype Present in Sera of TB Patients

**Figure 1:**
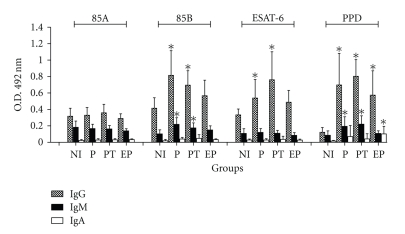
Levels of serum IgG (hatched box), IgM (black box) and IgA (empty box) to Ag85A, Ag85B, ESAT-6, and PPD were determined in serum of non-infected individuals (NI), pulmonary tuberculosis patients (P), pulmonary tuberculosis patients undergoing treatment (PT) and extra-pulmonary tuberculosis (EP). Results are presented as mean ± standard deviation. Differences between responses from TB patient groups and control groups were analyzed with nonparametric Kruskall-Wallis test. *Statistically significant differences in relation to NI group (*P* < .05).

To investigate the presence of specific anti-Ag85A, -Ag85B or -ESAT-6 antibodies in sera of TB patients with different clinical forms of the disease, ELISA were performed. Figure 1 shows the levels of specific IgG, IgM and IgA to mycobacterial antigens in sera of TB patients and healthy donors. The levels of anti-PPD IgG were significantly elevated in all tuberculosis patients compared to NI group. Furthermore, increased levels of IgG anti-Ag85B and anti-ESAT-6 were detected in P and PT groups compared to NI individuals. Interestingly, no significant titers of IgG anti-Ag85A were detected in studied patients. Levels of specific IgA antibodies to all antigens were very low and did not differ between the studied groups. In addition, only marginal anti-Ag85B and anti-PPD IgM levels were observed in the P and PT groups. 

**Figure 2:**
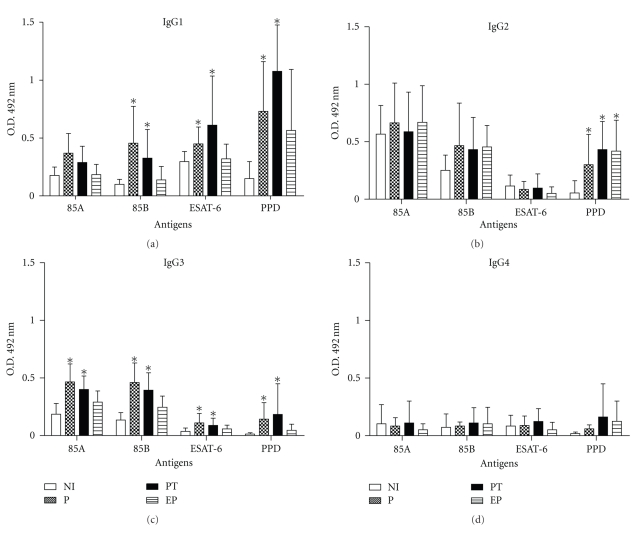
Levels of serum IgG1 (a), IgG2 (b), IgG3 (c) and IgG4 (d) to Ag85A, Ag85B, ESAT-6, and PPD were determined in serum of non-infected individuals (NI), pulmonary tuberculosis patients (P), pulmonary tuberculosis patients undergoing treatment (PT) and extra-pulmonary tuberculosis (EP). Results are presented as mean ± standard deviation. Differences between responses from TB patient groups and control groups were analyzed with nonparametric Kruskall-Wallis test. *Statistically significant differences in relation to NI group (*P* < .05).

Having observed elevated IgG levels to Ag85B and ESTA-6 antigens in patient sera, we decided to determine the IgG subclasses involved. The IgG subclass profile of TB patients was characterized predominantly by IgG1 responses to rAg85B and rESAT-6 on P and PT groups (Figure 2). Additionally, statistically significant levels of IgG3 to rAg85B, ESAT-6 and Ag85A were also detected in sera of PT and P group, however at lower levels. These results demonstrate the better performance of the Ag85B and ESAT-6 antigens compared to Ag85A to determine humoral responses in patients with active TB.

### 3.2. Proliferative Responses to Mycobacterial Antigens

**Figure 3:**
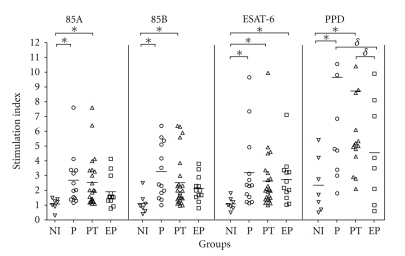
Lymphocyte proliferation in response to recombinant Ag85A, Ag85B and ESAT-6. Freshly isolated PBMC from non-infected individuals (NI), pulmonary TB patients (P), pulmonary TB patients in treatment (PT) and extra-pulmonary TB (EP) were cultured in the presence of Ag85A (25 *μ*g/ml), 85B (25 *μ*g/ml), PPD (25 *μ*g/ml) or ESAT-6 (50 *μ*g/ml) for 5 days and incorporation of [^3^H] thymidine was measured. Results are expressed as stimulation index (SI) mean of triplicate cultures. Horizontal bars indicate mean values. Differences between responses from TB patient groups and control groups were analyzed with nonparametric Kruskall-Wallis test. (*) Statistically significant differences in relation to NI group and (*δ*) in relation to EP group (*P* < .05).

In order to determine T cell-reactivity to the mycobacterial antigens tested, lymphoproliferative responses were measured in cells from tuberculosis patients and healthy individuals. As shown in Figure 3, proliferative responses upon stimulation with rESAT-6 were able to discriminate P, EP, and PT tuberculosis patients from healthy individuals. Regarding Ag85A and Ag85B, they were able to differentiate PT and P tuberculosis patients from noninfected individuals. Only when PPD was used as antigen, this assay was able to discriminate PT and P patients from individuals with extrapulmonary TB. PBMC of all individuals showed high cell proliferation after stimulation with PHA as a positive control (data not shown).

### 3.3. Cytokine Profile in Response to Recombinant Mycobacterial Antigens

**Figure 4:**
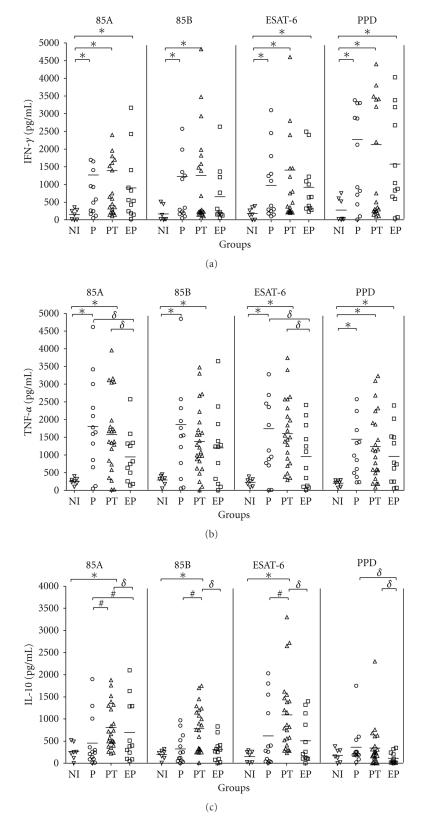
Individual IFN-*γ* (a), TNF-*α* (b), and IL-10 (c) production in response to recombinant Ag85A, Ag85B, and ESAT-6. Freshly isolated PBMC from non-infected individuals (NI), pulmonary TB patients (P), pulmonary TB patients in treatment (PT), and extra-pulmonary TB (EP) were cultured in the presence of Ag85A (25 *μ*g/ml), Ag85B (25 *μ*g/ml), PPD (25 *μ*g/ml), or ESAT6 (50 *μ*g/ml). Supernatants were harvested after five days and the cytokines measured by ELISA. Horizontal bars represent mean values. Differences between responses from TB patient groups and control groups were analyzed with nonparametric Kruskall-Wallis test. *Statistically significant differences in relation to NI group, (*δ*) in relation to EP group, and (#) in relation to P group (*P* < .05).

In order to evaluate the cytokines produced by P, EP, and PT patient cells to the mycobacterial recombinant antigens, IFN-*γ*, TNF-*α*, IL-10 and IL-4 were measured.  Figure 4 shows a significant production of IFN-*γ* after stimulation with Ag85A and ESAT-6 in the PT group (1285 ± 1571 and 1355 ± 976, resp.), P group (1268 ± 1722 and 974 ± 218, resp.), and EP group (903 ± 785 and 920 ± 2477, resp.) in comparison to the non-infected control group. This elevated production of IFN-*γ* in ESAT6-stimulated PBMC of TB patients correlated with positive response to ESAT6 in the proliferation assays (Spearman test; PT group *r* = 0.5158, *P* = .0118; P group *r* = 0.5659, *P* = .0438; EP group *r* = 0.6224, *P* = .0307). Moreover, in response to Ag85B, only PT (1206 ± 2087) and P (1227 ± 799) patients produced significant levels of IFN-*γ* compared to healthy individuals. The PT groups were the higher producers of IFN-*γ* in response to all recombinant antigens; however no difference was observed within this group among the different antigens used. All TB patients produced high levels of IFN-*γ* to PPD, and this result is in agreement with the complex antigenic mixture of PPD.

Regarding TNF-*α* production, we observed that PT and  P groups produced significant levels of this cytokine when stimulated with Ag85A, Ag85B or ESAT-6. However, no recombinant antigens tested were able to induce greater levels of TNF-*α* in EP group compared to healthy individuals. Furthermore, only Ag85A or ESAT6 antigens induced production of higher amounts of TNF-*α* by PT and P cells that allowed discrimination between these groups from EP group. Additionally, all patients produced high levels of TNF-*α* to PPD.

As for IL-10, a regulatory cytokine, we observed that the group of TB patients who had initiated chemotherapy (PT) produced higher amounts of IL-10 compared to P or EP groups when Ag85A, Ag85B and ESAT-6 were tested. These values were significant to differentiate PT from P and EP groups. None of the tested recombinant antigens or PPD induced detectable production of IL-4 by PBMC of all individuals tested (data not shown).

## 4. Discussion

For the development of new vaccines and diagnostic reagents, there is an urgent need for assessment of immune responses to *M. tuberculosis* antigens in areas of TB endemicity. In the present study T-cell and antibody responses to recombinant Ag85A, Ag85B or ESAT-6 were investigated in Brazilian patients with pulmonary or extra-pulmonary TB and patients undergoing treatment compared to non-infected individuals. Several studies have detected antibodies in sera of patients with active TB against a variety of *M. tuberculosis* antigens [20–22]. Herein, patients with active disease or undergoing >2 months of treatment presented elevated levels of IgG anti-ESAT-6 and anti-Ag85B but not to Ag85A. High levels of antibodies against filtered *M. tuberculosis* antigens in the first two months of chemotherapy have been associated with intense stimulation of the humoral response by antigens released from killed bacteria combined with the disappearance of circulating mycobacterial antigens so that specific antibodies are no longer trapped in the immune complexes [23]. Therefore, large amounts of IgG antibodies against secreted Ag85B and ESAT-6 antigens appear to be associated with viable and metabolically active bacilli. Little attention has been given to the subclasses involved in TB [24]. In our study, the analysis of IgG subclasses to the mycobacterial recombinant antigens revealed the predominance of IgG1 but not IgG2 and IgG4 to ESAT-6 and Ag85B in sera of patients of the P or PT group. However, we also detected significant levels of IgG3 against Ag85A, Ag85B and ESAT-6 in these groups of patients. Our results are consistent with other studies that also observed predominance of IgG1 antibodies in the sera of patients with active TB [19, 24, 25]. Since Ag85A and Ag85B share around 77% of amino acids identity, one could expect them to have common immunodominant epitopes. Despite pronounced sequence homology among these Ag85 members, D'Souza et al. [26] have shown that different Ag85-specific immunodominant T-cell epitopes were identified in BALB/c and C57BL/6 mouse strains. These differences in MHC-restriction during Ag85A and Ag85B epitope mapping might be one of the reasons why we did not observed significant levels of anti-Ag85A IgG in our TB patients. Similarly, Van Vooren et al. [27] suggested that Ag85B was the most useful component of the Ag85 complex for serodiagnosis of the active form of TB.

Recently, commercial immunodiagnostic tests for TB have been introduced. These tests are based on the *M. tuberculosis* ESAT-6 and culture filtrate protein 10 (CFP-10) and include a whole-blood IFN-*γ* ELISA (QuantiFERON-TB Gold, Cellestis Ltd, Victoria, Australia) and an ELISPOT assay (T-SPOT.TB, Oxford Immunotec, Oxfordshire, UK). Both tests have shown promising results in the detection of latent TB and the potential use for differential diagnoses of active tuberculosis [28, 29]. However, the sensitivities and specificities of these assays vary among the different populations studied, due mostly to the different HLA genetic backgrounds, the prevalence of TB infection, and the coverage of *M. bovis* BCG vaccination [30]. Furthermore, there is a need to develop new diagnostic tools to detect extra-pulmonary TB and sputum negative cases. In our study, pulmonary or extra-pulmonary TB patients and individuals undergoing chemotherapy responded to ESAT-6 as evaluated by lymphoproliferative responses or by IFN-*γ* production determined in the supernatants of stimulated PBMC. Additionally, Ag85A was also recognized by all TB patient cells compared to non-infected individuals as measured by IFN-*γ* secretion. This data demonstrates that ESAT-6 and Ag85A are recognized by T cell from many tuberculosis patients undergoing distinct periods of clinical disease and is consistent with Ulrichs et al. [15] findings by which PBMCs from tuberculosis patients, but not healthy donors, respond to ESAT-6. Furthermore, as described previously by Antas et al. [31], the Ag85A and Ag85B proteins were also recognized by PBMC of pulmonary tuberculosis patients and individuals undergoing treatment measured by elevated proliferation and IFN-*γ* production. Regarding TNF-*α*, clinical studies have associated the use of TNF-blockers with progression from latent tuberculosis infection to disease [32]. Further, Caccamo et al. [33] have reported high percentage of CD4+ T cells expressing IFN-*γ*/IL-2/TNF-*α* in active TB patients and it seems to be associated with live bacterial loads, as indicated by the decrease in frequency of multifunctional T cells in TB patients after completion of antimycobacterial therapy. In our study, we have observed elevated levels of TNF-*α* to Ag85A, Ag85B, and ESAT-6 in patients with pulmonary tuberculosis or undergoing treatment but not in extra-pulmonary TB patients. However, only Ag85A and ESAT-6 antigens were able to discriminate PT and P patients from EP. This data might be associated to differential production of TNF-*α* by CD4+, and CD8+ T cells and the compartmentalization of immune response at site of disease. Marei et al. [34] demonstrated a differential expression of IFN-*γ* and TNF-*α* in CD4+ T cells and CD8+ T cells after stimulation with ESAT-6. In their study, CD4+ T cells are the main producer of TNF-*α* while IFN-*γ* was produced by either CD4+ or CD8+ T cells. In addition, other studies have provided evidence for compartmentalization of Th1 cytokines at the site of disease in humans [35, 36]. We suggest that compartmentalization of the immune response in EP patients can lead to sequestration of TNF-*α*  producing cells at the disease site and it contributes to reduced production of this cytokine in peripheral blood in response to Ag85A, Ag85B and ESAT-6. Herein, our results suggest that Ag85A and ESAT-6 are able to differentiate P, PT and EP patients from healthy individuals by IFN-*γ* production and from P and PT groups to EP individuals by production of TNF-*α*. In a recent study, it was reported that similar levels of cytokine and antibody responses to *M. tuberculosis* ESAT-6/CFP-10 fusion protein were detected in PPD+ and PPD− groups from an endemic area of Juiz de Fora, Minas Gerais, Brazil [19]. Herein, the NI group (PPD−) used also lives in a TB endemic area in Brazil and probably has been exposed to multiple forms of environmental mycobacteria. However, to confirm the diagnostic potential of these antigens further studies using BCG vaccinated controls (PPD+) are required. 

Antibodies conventionally are considered to play little role in defence against mycobacteria, and the function of antibodies in pathogenesis is yet to be determined. Macrophages in which mycobacteria resides, and multiply have high affinity receptors (Fc*γ*1 and Fc*γ*3) for IgG1 and IgG3 antibodies, and the presence of IgG1 and IgG3 antibodies may enhance bacterial uptake and clearance of pathogen via the Fc receptor [37]. Hussain et al. [38] reported that opsonizing antibodies upregulate macrophage proinflammatory cytokines TNF-*α* and IL-6 in mycobacterial-stimulated macrophages thus suggesting a role for this isotype in TB, since TNF-*α* synergizes with IFN-*γ* in its tuberculostatic activity. Since IgG responses against proteins are T cell dependent, antigen recognition by IgG isotypes implies that helper T cells also recognize these mycobacterial antigens. Further, IFN-*γ* produced by Th1 cells induces murine IgG2a and IgG2b and human IgG1 and IgG3 [39, 40]. Human IgG1 and IgG3 counterparts of murine IgG2a and IgG2b share the ability to fix complement and function as opsonins. In this study, we observed that the increased IgG1 was coincident with augmented levels of IFN-*γ* and TNF-*α* detected in PBMCs of patients with active TB and individuals undergoing treatment stimulated with Ag85B and ESAT-6. These results suggest a possible correlation of IgG1 production with Th1 and inflammatory response in TB. 

Interestingly, IL-10 production was elevated only in patients under treatment in response to Ag85, Ag86, and ESAT-6. In this study, IL-10 levels could differentiate individuals undergoing chemotherapy from pulmonary or extra-pulmonary patients. Priya et al. [41] have shown that TB patients from India at the beginning of chemotherapy produced similar levels of IFN-*γ* and IL-10 to Ag85A and the ratio of IFN/IL-10 increases after successful treatment. Furthermore, Meyaard et al. [42] demonstrated that IL-12 is able to induce T cells to produce IL-10 and suggest that IL-10 is a negative regulator of IL-12- induced T cell response. In another study testing TB patients, Priya et al. [43] observed that high levels of IL-10 detected in active TB decreased in patients considered cured. These results and our data led us to hypothesize that elevated production of IL-10 encountered in PT group is probably a modulatory effect in response to IL-12 and IFN-*γ* production and can be associated to the regulation of immune response. 

Th2 responses characterized by inteleukin-4 (IL-4) production have been associated with a lack of protection in TB [44]. In our study, IL-4 levels in response to mycobacterial recombinant antigens were not detected in all groups analyzed. These results are in accordance with others that show that PBMC from TB patients do not produce significant amounts of IL-4 [45–47]. These results confirm the polarization of immune response to recombinant antigens to Th1 profile characterized by the production of high levels of IFN-*γ* and TNF-*α* and no IL-4.

Finally, TB causes a staggering burden of mortality worldwide, killing an estimated 1.9 million persons annually. Effective treatment of tuberculosis in developing countries is hampered by the cost of antituberculosis drugs, inability to ensure completion of therapy, and rising drug resistant rates. Vaccination is the most cost-effective strategy to control and eventual elimination of tuberculosis. The current BCG vaccine provides some degree of protection against the most severe manifestations of childhood tuberculosis. However, protection is incomplete, and BCG vaccine does not reduce TB rates in adults. In fact, MVA85A, a recombinant modified vaccinia virus Ankara expressing Ag85A, is the first candidate TB subunit vaccine to enter human trials since BCG was first introduced over 80 years ago. More recently, Dissel et al. [48] demonstrated that vaccination of human naïve volunteers with adjuvanted Ag85B-ESAT-6 subunit vaccine elicited strong antigen-specific T-cell responses. Since a basic principle for selecting novel antigen candidates for designing a TB subunit vaccine is based on their ability to induce a protective Th1 response [16], our study also confirmed the value of Ag85A, Ag85B and ESAT-6 as potential vaccine candidates based upon specific T cell responses measured by IFN-*γ* and TNF-*α* production in all studied patients.

## 5. Conclusions

Currently, there are no accurate surrogate biomarkers of protective immunity and diagnoses in TB but clearly host defense against TB depends critically on Th1 responses and IFN-*γ* production. In this study, we have shown that Ag85A and ESAT-6 are antigens able to differentiate pulmonary, extra-pulmonary and tuberculosis patients undergoing chemotherapy from healthy individuals by IFN-*γ* production and pulmonary and under treatment patients from extra-pulmonary TB by TNF-*α*. Therefore, not only IFN-*γ* production but also TNF-*α* to Ag85A and ESAT-6 could be used as biomarkers for the clinical status of TB patients while IL-10 could be useful monitoring TB successful treatment. Finally, the Th1 cytokine profile induced in PBMC of TB patients by all tested antigens reinforces their position as potential vaccine candidates.

## Figures and Tables

**Table 1:** Clinical characteristics of TB patients and controls in this study.

Groups	No. of subjects	TST	Males/females	Age (mean ± SD)	Age range
Non-infected (NI)	7	−	05/02	40.1 ± 9.5	28–55
TB patients under treatment (PT)*	25	+	14/11	39.8 ± 14.6	22–76
Pulmonary TB untreated (P)	13	+	09/04	41.0 ± 15.7	19–69
Extra-pulmonary TB (EP)	12	+	08/04	41.1 ± 16.5	21–76
(i) Pleural	6				
(ii) Miliary	5				
(iii) Intestinal	1				

*The treatment consisted of Rifampicin (10–20 mg/kg/day), Isoniazide (10–20 mg/kg/day), and Piraminazide (30–50 mg/kg/day). PT patients were undergoing 1–3 months of chemotherapy. TST tuberculin skin test.
